# Supplementary material for: Imp/IGF2BP levels modulate individual neural stem cell growth and division through myc mRNA stability
Source: eLife. 2020 Jan 14;9:e51529. doi: 10.7554/eLife.51529 (PMC7025822; doi:10.7554/eLife.51529)

BrainImp:

Analysis of Imp targets in *D. melanogaster* larval brain

Contents

DESCRIPTION **2**
Project: Imp levels modulate neural stem cell growth and division . . . 2
Analysis Overview . . . 2

REQUIRED SOFTWARE AND LIBRARIES **3**
R libraries . . . 3

PRE-PROCESSING: FROM RAW READS TO GENE COUNTS **4**
Defining target genome and annotation . . . 4
Download . . . 4
Calculate gene lengths . . . 4
Annotate gene overlaps . . . 5
Mapping reads to genome . . . 6
Make STAR index . . . 6
Map reads to genome with STAR . . . 6
Counting reads per gene . . . 8

ANALYSING IMP TARGETS **9**
Make a count table with all the samples . . . 9
Rank Imp targets . . . 9
DESeq analysis . . . 11
Annotate overlapping genes . . . 13
GO term analysis . . . 13
Make Figure 2 . . . 15

DESCRIPTION

Project: Imp levels modulate neural stem cell growth and division

This is a supplementary document to the Samuel's et al. manuscript ***"Imp/IGF2BP levels modulate individual neural stem cell growth and division through myc mRNA stability"***, detailing the bioinformatic analysis of the Imp RIP-seq and RNA-seq data presented.

Analysis Overview

The following **command line tools** have been used in the below analysis:

- **STAR aligner** (2.5.3a) to map reads in fastq files to the *D. melanogaster* genome (BDGP6.22.97)
- **htseq-count** (0.11.2) to assign mapped reads to the *D. melanogaster* annotation (BDGP6.22.97)

The following **R libraries** have been used in the below analysis:

- **tidyverse** (1.2.1) for general data manipulation and plotting
- **rtracklayer** (1.44.3) for reading gtf annotation files
- **GenomicRanges** (1.36.0) to analyse genomic annotations
- **DESeq2** (1.24.0) for differential expression analysis
- **GO.db** (3.8.2) to retrieve GO terms
- **biomaRt** (2.40.4) to map genes to GO terms

This is a document created with **RStudio Notebook**.

REQUIRED SOFTWARE AND LIBRARIES

R libraries

```r
#-#-#-#-#-#-#-#-#-#-#-#-#-#-#-#-#-#-#-#-#-#-#-#-#-#-#-#-#-#-#-#-#-#-#-#-#-#-#-#-#-#-#-#-#-
# Load required R libraries
#-#-#-#-#-#-#-#-#-#-#-#-#-#-#-#-#-#-#-#-#-#-#-#-#-#-#-#-#-#-#-#-#-#-#-#-#-#-#-#-#-#-#-#-#-

library(tidyverse)
library(rtracklayer)
library(GenomicRanges)
library(DESeq2)
library(GO.db)
library(biomaRt)
```

PRE-PROCESSING: FROM RAW READS TO GENE COUNTS

In this step, reads are mapped to the target genome and mapped reads assigned to gene annotations.

Defining target genome and annotation

Download

Download *D. melanogaster* genome and annotation from ENSEMBL.

```
#-#-#-#-#-#-#-#-#-#-#-#-#-#-#-#-#-#-#-#-#-#-#-#-#-#-#-#-#-#-#-#-#-#-#-#-#-#-#-#-#-#-#-#-#-
# Fly Genome
#-#-#-#-#-#-#-#-#-#-#-#-#-#-#-#-#-#-#-#-#-#-#-#-#-#-#-#-#-#-#-#-#-#-#-#-#-#-#-#-#-#-#-#-#-

cd "$PROJECTHOME"
mkdir -p ./Preprocessing/Genome/DNA
cd ./Preprocessing/Genome/DNA/

wget ftp://ftp.ensembl.org/pub/release-97/fasta/drosophila_melanogaster/dna/Drosophila_melanogaster.BDG[illegible]
gunzip Drosophila_melanogaster.BDGP6.22.dna.chromosome.*.fa.gz
cat Drosophila_melanogaster.BDGP6.22.dna.chromosome.*.fa > Drosophila_melanogaster.BDGP6.22.dna.fa
gzip Drosophila_melanogaster.BDGP6.22.dna.chromosome.*.fa

#-#-#-#-#-#-#-#-#-#-#-#-#-#-#-#-#-#-#-#-#-#-#-#-#-#-#-#-#-#-#-#-#-#-#-#-#-#-#-#-#-#-#-#-#-
# Fly Annotation
#-#-#-#-#-#-#-#-#-#-#-#-#-#-#-#-#-#-#-#-#-#-#-#-#-#-#-#-#-#-#-#-#-#-#-#-#-#-#-#-#-#-#-#-#-

cd "$PROJECTHOME"
mkdir -p ./Preprocessing/Genome/GeneSets
cd ./Preprocessing/Genome/GeneSets/

wget ftp://ftp.ensembl.org/pub/release-97/gtf/drosophila_melanogaster/Drosophila_melanogaster.BDGP6.22.[illegible]
gunzip Drosophila_melanogaster.BDGP6.22.97.chr.gtf.gz
```

Calculate gene lengths

Calculate gene lengths based on the lengths of its transcripts. Transcript length is calculated as a sum of its exon lengths.

```
#-#-#-#-#-#-#-#-#-#-#-#-#-#-#-#-#-#-#-#-#-#-#-#-#-#-#-#-#-#-#-#-#-#-#-#-#-#-#-#-#-#-#-#-#-
# Calculate gene lengths (based on transcript exon lengths)
#-#-#-#-#-#-#-#-#-#-#-#-#-#-#-#-#-#-#-#-#-#-#-#-#-#-#-#-#-#-#-#-#-#-#-#-#-#-#-#-#-#-#-#-#-

# -- Read annotation (exons only) using rtracklayer import function ---------------------

gtf <- rtracklayer::import( file.path( project.home, '/Preprocessing/Genome/GeneSets/',
                                       'Drosophila_melanogaster.BDGP6.22.97.chr.gtf' ),
                              feature.type="exon")

# -- Calculate tx and gene length summaries from exon widths ----------------------------

# -- Convert to tibble
exons <- as_tibble( as.data.frame( gtf ) )
```

```r
# -- Calculate transcript lengths by summing up exons
tx.info <- exons %>%
  select(gene_id, gene_name, transcript_id, width ) %>%
  group_by( gene_id, transcript_id ) %>%
  summarise( n.exons=n(), tx.length=sum(width))

# -- Calculate gene lengths from transcript lengths
gene.info <- tx.info %>% group_by( gene_id ) %>% summarise(
  n.tx=n(),
  length.max=max(tx.length),
  length.min=min(tx.length),
  length.mean=mean(tx.length),
  length.median=median(tx.length)
  )

# -- Save
save(tx.info, gene.info, file= file.path(
  project.home, '/Preprocessing/Genome/GeneSets/', 'Dm.BDGP6.22.97.gene-tx-lengths.RData' ))
```

Annotate gene overlaps

Annotated which ncRNAs are inside other genes (coding or non-coding). Many short ncRNAs are generated from introns of protein coding genes. This might result in them getting 'false' counts when reads are assigned to genes (see below) if there is a small proportion of intronic signal retained from the 'host' protein coding genes. The idea here to flag such instances, so that they can be considered separately.

```r
#-#-#-#-#-#-#-#-#-#-#-#-#-#-#-#-#-#-#-#-#-#-#-#-#-#-#-#-#-#-#-#-#-#-#-#-#-#-#-#-#-
# Annotate gene overlaps
#-#-#-#-#-#-#-#-#-#-#-#-#-#-#-#-#-#-#-#-#-#-#-#-#-#-#-#-#-#-#-#-#-#-#-#-#-#-#-#-#-

# -- Read annotation (genes only) using rtracklayer import function ---------------------

gtf <- rtracklayer::import( file.path( project.home, '/Preprocessing/Genome/GeneSets/',
                                       'Drosophila_melanogaster.BDGP6.22.97.chr.gtf' ),
                              feature.type="gene")

# -- Find gene overlaps -----------------------------------------------------------------

gene.overlap <- GenomicRanges::findOverlaps(gtf, gtf, ignore.strand=FALSE)

overlap.info <- tibble( query.gene.id        = gtf[ queryHits(gene.overlap) ]$gene_id,
                        query.gene.name      = gtf[ queryHits(gene.overlap) ]$gene_name,
                        query.gene.biotype   = gtf[ queryHits(gene.overlap) ]$gene_biotype,
                        target.gene.id       = gtf[ subjectHits( gene.overlap ) ]$gene_id,
                        target.gene.name     = gtf[ subjectHits( gene.overlap ) ]$gene_name,
                        target.gene.biotype  = gtf[ subjectHits( gene.overlap ) ]$gene_biotype )

genes.with.overlaps <- overlap.info %>% filter(query.gene.id != target.gene.id)

# -- Save
save(genes.with.overlaps, file = file.path(
  project.home, '/Preprocessing/Genome/GeneSets/', 'Dm.BDGP6.22.97.gene-overlap.info.RData' ))
```

Mapping reads to genome

Map reads to D.melanogaster genome using STAR (2.5.3a).

Make STAR index

Make an index for STAR mapping using STAR genomeGenerate.

```
#-#-#-#-#-#-#-#-#-#-#-#-#-#-#-#-#-#-#-#-#-#-#-#-#-#-#-#-#-#-#-#-#-#-#-#-#-#-#-#-#-#-#-#-#-
# Make STAR index
#-#-#-#-#-#-#-#-#-#-#-#-#-#-#-#-#-#-#-#-#-#-#-#-#-#-#-#-#-#-#-#-#-#-#-#-#-#-#-#-#-#-#-#-#-

cd "$PROJECTHOME"
mkdir -p ./Preprocessing/Genome/STAR
cd ./Preprocessing/Genome/

STAR --runThreadN 3 --runMode genomeGenerate --genomeDir ./STAR
   --genomeFastaFiles ./DNA/Drosophila_melanogaster.BDGP6.22.dna.fa \
   --alignSJoverhangMin 8 --outFileNamePrefix Dmelanogaster-BDGP6.22.97 \
   --sjdbGTFfile ./GeneSets/Drosophila_melanogaster.BDGP6.22.97.chr.gtf \
   --sjdbOverhang 100 --limitGenomeGenerateRAM 12000000000 \
   --genomeSAindexNbases 10 --genomeSAsparseD 4
```

Map reads to genome with STAR

Map reads to genome with STAR using STAR alignReads.

```
#-#-#-#-#-#-#-#-#-#-#-#-#-#-#-#-#-#-#-#-#-#-#-#-#-#-#-#-#-#-#-#-#-#-#-#-#-#-#-#-#-#-#-#-#-
# Align Imp RIP-seq data
#-#-#-#-#-#-#-#-#-#-#-#-#-#-#-#-#-#-#-#-#-#-#-#-#-#-#-#-#-#-#-#-#-#-#-#-#-#-#-#-#-#-#-#-#-

for file in $(basename --  "$PROJECTHOME"/Data/brain-ripseq-imp-*fastq); do
  outdir="${file/.fastq/}"
  OUTDIR="$PROJECTHOME"/Preprocessing/Mapping/$outdir/
  echo ""
  echo "Input file: $PROJECTHOME/Data/$file"
  echo "Output directory: $OUTDIR"
  if [ -d "$OUTDIR" ]
  then
      echo 'Error: Directory exists. SKIPPING!'
      continue
  else
      echo 'Running STAR...'
      mkdir -p "$OUTDIR"
      STAR --runMode alignReads --runThreadN 3 --outSAMtype BAM Unsorted \
           --genomeDir "$PROJECTHOME"/Preprocessing/Genome/STAR \
           --readFilesIn "$PROJECTHOME"/Data/$file --outFileNamePrefix "$OUTDIR"/ \
           --sjdbGTFfile "$PROJECTHOME"/Preprocessing/Genome/GeneSets/Drosophila_melanogaster.BDGP6.22.[illegible]
  fi
done
gzip "$PROJECTHOME"/Data/brain-ripseq-imp-*fastq

#-#-#-#-#-#-#-#-#-#-#-#-#-#-#-#-#-#-#-#-#-#-#-#-#-#-#-#-#-#-#-#-#-#-#-#-#-#-#-#-#-#-#-#-#-
```

```
# Align RNA-seq data
#-#-#-#-#-#-#-#-#-#-#-#-#-#-#-#-#-#-#-#-#-#-#-#-#-#-#-#-#-#-#-#-#-#-#-#-#-#-#-#-#-#-#-#-#-

for file in $(basename --  "$PROJECTHOME"/Data/brain-impGFPbackground-rnaseq-*fastq); do
  outdir="${file/-impGFPbackground/}"
  outdir="${outdir/.fastq/}"
  OUTDIR="$PROJECTHOME"/Preprocessing/Mapping/$outdir/
  echo ""
  echo "Input file: $PROJECTHOME/Data/$file"
  echo "Output directory: $OUTDIR"
  if [ -d "$OUTDIR" ]
  then
      echo 'Error: Directory exists. SKIPPING!'
      continue
  else
      echo 'Running STAR...'
      mkdir -p "$OUTDIR"
      STAR --runMode alignReads --runThreadN 3 --outSAMtype BAM Unsorted \
           --genomeDir "$PROJECTHOME"/Preprocessing/Genome/STAR \
           --readFilesIn "$PROJECTHOME"/Data/$file --outFileNamePrefix "$OUTDIR"/ \
           --sjdbGTFfile "$PROJECTHOME"/Preprocessing/Genome/GeneSets/Drosophila_melanogaster.BDGP6.22.[illegible]
  fi
done
gzip "$PROJECTHOME"/Data/brain-impGFPbackground-rnaseq-*fastq
```

Counting reads per gene

Count reads per gene using different annotation feature types using HTSeq framework version 0.11.2. Gene counts are based on 'exon' feature type in this project. The other types ('gene', 'CDS', 'three_prime_utr', 'five_prime_utr') are determined just to identify differences in intron, coding region, and UTR usage, and are only used in subset of analyses.

```
#-#-#-#-#-#-#-#-#-#-#-#-#-#-#-#-#-#-#-#-#-#-#-#-#-#-#-#-#-#-#-#-#-#-#-#-#-#-#-#-#-#-#-#-#-
# Count reads per gene using different annotation feature types
#-#-#-#-#-#-#-#-#-#-#-#-#-#-#-#-#-#-#-#-#-#-#-#-#-#-#-#-#-#-#-#-#-#-#-#-#-#-#-#-#-#-#-#-#-

mkdir -p "$PROJECTHOME"/Preprocessing/Counting/

for type in 'exon' 'gene' 'CDS' 'three_prime_utr' 'five_prime_utr'; do
  echo ""
  echo ""
  echo "Running htseq-count for feature type $type"
  for INDIR in $(basename --  "$PROJECTHOME"/Preprocessing/Mapping/*/); do
    nametype=$type
    if [ "$(echo $nametype | grep 'three_prime_utr')" != ""  ]; then nametype='3UTR'; fi
    if [ "$(echo $nametype | grep 'five_prime_utr')" != ""  ]; then nametype='5UTR'; fi
    inputfile="$PROJECTHOME"/Preprocessing/Mapping/$INDIR/Aligned.out.bam
    outputfile="$PROJECTHOME"/Preprocessing/Counting/$INDIR.stranded.$nametype.tsv
    echo ""
    echo "Input file: $inputfile"
    echo "Output file: $outputfile"
    htseq-count --additional-attr gene_name -f bam -s yes -t $type -i \
            gene_id $inputfile
            "$PROJECTHOME"/Preprocessing/Genome/GeneSets/Drosophila_melanogaster.BDGP6.22.97.chr.gt
            > $outputfile
  done
done
```

ANALYSING IMP TARGETS

In this step, we look at Imp targets.

Make a count table with all the samples

Prepare a count table tibbles for different assigment types (exons, genes, CDS, . . .). Combine RIP-seq and RNA-seq into one table.

```
#-#-#-#-#-#-#-#-#-#-#-#-#-#-#-#-#-#-#-#-#-#-#-#-#-#-#-#-#-#-#-#-#-#-#-#-#-#-#-#-#-#-#-#-#-
# Combine RIP-seq and RNA-seq count tables into one R object
#-#-#-#-#-#-#-#-#-#-#-#-#-#-#-#-#-#-#-#-#-#-#-#-#-#-#-#-#-#-#-#-#-#-#-#-#-#-#-#-#-#-#-#-#-

mapping.dir <- 'Preprocessing/Counting/'

# -- Helper functions ------------------------------------------------------------------

MakeCountTable <- function(path=".", pattern=".stranded.exon.tsv",
                           skip.last=5, sub=c('brain-', '.tsv')) {
  # Find count files
  files <- list.files( path=path, pattern = pattern)
  # Read count files.
  # Note that last 5 lines in input files contain general htseq-count info, no gene counts.
  count.tbl <- lapply( files, function(file) {
    tbl <- readr::read_tsv( file.path( project.home, mapping.dir, file ), col_names = F )
    tbl <- tbl %>% dplyr::slice( 1:( (tbl %>% count())[[1]] - skip.last ))
    to.sub <- paste0(paste0(paste0( '(', sub ), ')'),collapse = "|")
    name <- gsub( '-', '\\.', gsub(to.sub, '', file) )
    tbl <- tbl %>% rename_at(1:3, funs( c('gene.id', 'gene.name', name) ))
    return(tbl)
  })
  # Join datasets
  data <- count.tbl %>% purrr::reduce(full_join)
  colnames(data) <- make.unique(names(data))
  # Return
  return(data)
}

# -- Make one big count table with all RIP-seq and RNA-seq replicates ------------------

counts <- MakeCountTable(
  path=file.path( project.home, mapping.dir ),
  pattern='brain-[a-z]{3}seq-[a-z]{,3}[-]?[1-3].stranded.(exon|gene|CDS|3UTR|5UTR).tsv')

save(counts, file=file.path(project.home, mapping.dir, 'counts.RData') )
rm(list=ls())
```

Rank Imp targets

Library size and transcript-length normalisation. Ranking.

```
#-#-#-#-#-#-#-#-#-#-#-#-#-#-#-#-#-#-#-#-#-#-#-#-#-#-#-#-#-#-#-#-#-#-#-#-#-#-#-#-#-#-#-#-#-
# Normalise by library size and gene length
```

```r
#-#-#-#-#-#-#-#-#-#-#-#-#-#-#-#-#-#-#-#-#-#-#-#-#-#-#-#-#-#-#-#-#-#-#-#-#-#-#-#-#-#-#-#-#-

mapping.dir <- 'Preprocessing/Counting/'

# -- Helper functions -------------------------------------------------------------------

NormalizeLibrary <- function(counts) { return(10^6*counts/sum(counts)) }

# -- Load data --------------------------------------------------------------------------

# -- Load count tibble (counts) calculated above
load(file.path(project.home, mapping.dir, 'counts.RData') )

# -- Extract exon counts only
gene.counts <- counts %>% select(gene.id, gene.name, contains('exon'))

# -- Change column names a little
gene.counts <- gene.counts %>%
  rename_at(vars(ends_with(".stranded.exon")),
            funs(str_replace(., ".stranded.exon", "" )) )  %>%
  rename_at(vars(matches("seq.")), funs(str_replace(., "seq.", "." )) ) %>%
  rename_at(vars(matches("imp.")), funs(str_replace(., "imp.", "." )) ) %>%
  rename_at(vars(matches("rip.")), funs(str_replace(., "rip.", "imp" )) )

# -- Normalise by library size ----------------------------------------------------------

gene.counts <- gene.counts %>% mutate(
  imp.1.norm.lib=NormalizeLibrary(imp.1),
  imp.2.norm.lib=NormalizeLibrary(imp.2),
  imp.3.norm.lib=NormalizeLibrary(imp.3),
  rna.1.norm.lib=NormalizeLibrary(rna.1),
  rna.2.norm.lib=NormalizeLibrary(rna.2),
  rna.3.norm.lib=NormalizeLibrary(rna.3)
)

# -- Normalise by gene length -----------------------------------------------------------

# -- Load gene lengths (gene.info) calculated above
load(file.path( project.home, '/Preprocessing/Genome/GeneSets/',
                'Dm.BDGP6.22.97.gene-tx-lengths.RData' ))

# -- Attach gene lengths
gene.counts <- left_join(gene.counts, gene.info, by=c("gene.id" = "gene_id"))

# -- Divide library size normalised counts with gene mean length
gene.counts <- gene.counts %>% mutate(
  imp.1.norm.lib.len=imp.1.norm.lib/length.mean,
  imp.2.norm.lib.len=imp.2.norm.lib/length.mean,
  imp.3.norm.lib.len=imp.3.norm.lib/length.mean,
  rna.1.norm.lib.len=rna.1.norm.lib/length.mean,
  rna.2.norm.lib.len=rna.2.norm.lib/length.mean,
  rna.3.norm.lib.len=rna.3.norm.lib/length.mean
)
```

```
# -- Calculate mean normalised count ----------------------------------------------------

gene.counts <- gene.counts %>% rowwise() %>%
  mutate( imp.norm.lib.mean=mean(c( imp.1.norm.lib, imp.2.norm.lib, imp.3.norm.lib )),
          rna.norm.lib.mean=mean(c( rna.1.norm.lib, rna.2.norm.lib, rna.3.norm.lib )),
          imp.norm.lib.len.mean=
            mean(c( imp.1.norm.lib.len, imp.2.norm.lib.len, imp.3.norm.lib.len )),
          rna.norm.lib.len.mean=
            mean(c( rna.1.norm.lib.len, rna.2.norm.lib.len, rna.3.norm.lib.len )) ) %>%
  ungroup()

# -- Imp relative to RNAseq -------------------------------------------------------------

gene.counts <- gene.counts %>% mutate(
  imp.over.rna.lib.mean= imp.norm.lib.mean/rna.norm.lib.mean )

#-#-#-#-#-#-#-#-#-#-#-#-#-#-#-#-#-#-#-#-#-#-#-#-#-#-#-#-#-#-#-#-#-#-#-#-#-#-#-#-#-#-#-#-#-
# Rank
#-#-#-#-#-#-#-#-#-#-#-#-#-#-#-#-#-#-#-#-#-#-#-#-#-#-#-#-#-#-#-#-#-#-#-#-#-#-#-#-#-#-#-#-#-

# -- Rank -------------------------------------------------------------------------------

gene.counts <- gene.counts %>%
  mutate(imp.norm.lib.mean.rank=dense_rank(dplyr::desc(imp.norm.lib.mean)),
         rna.norm.lib.mean.rank=dense_rank(dplyr::desc(rna.norm.lib.mean)),
         imp.norm.lib.len.mean.rank=dense_rank(dplyr::desc(imp.norm.lib.len.mean)),
         rna.norm.lib.len.mean.rank=dense_rank(dplyr::desc(rna.norm.lib.len.mean)),
         imp.over.rna.lib.mean.rank=dense_rank(dplyr::desc(imp.over.rna.lib.mean)))

# -- Rank: without very genes with minimal counts ---------------------------------------

gene.expressed <- gene.counts %>% rowwise() %>%
  mutate( rna.sum=sum(rna.1, rna.2, rna.3) ) %>%
  ungroup() %>% filter(rna.sum >= 10) %>% select( -rna.sum)

gene.expressed <- gene.expressed %>%
  mutate(imp.over.rna.lib.mean.expr.rank=dense_rank(dplyr::desc(imp.over.rna.lib.mean))) %>%
  select(gene.id, gene.name, imp.over.rna.lib.mean.expr.rank)

gene.counts <- left_join(gene.counts, gene.expressed) %>%
  arrange(imp.over.rna.lib.mean.expr.rank)

# -- Save -------------------------------------------------------------------------------

target.dir <- 'ImpTargets/'
dir.create(file.path(project.home, target.dir))
save(gene.counts, file=file.path(project.home, target.dir, 'counts.ranked.RData') )
```

DESeq analysis

```r
#-#-#-#-#-#-#-#-#-#-#-#-#-#-#-#-#-#-#-#-#-#-#-#-#-#-#-#-#-#-#-#-#-#-#-#-#-#-#-#-#-#-#-#-#-
# Imp targets relative to baseline expression: analysis with DESeq2
#-#-#-#-#-#-#-#-#-#-#-#-#-#-#-#-#-#-#-#-#-#-#-#-#-#-#-#-#-#-#-#-#-#-#-#-#-#-#-#-#-#-#-#-#-

target.dir <- 'ImpTargets/'

# -- Prepare DESeqDataSet object ---------------------------------------------------

# -- Load gene.counts
load(file.path(project.home, target.dir, 'counts.ranked.RData') )

# -- Prepare count table and condition table
count.table           <- data.frame( gene.counts %>%
                                     select( rna.1, rna.2, rna.3, imp.1, imp.2 , imp.3) )
rownames(count.table) <- gene.counts$gene.id
condition             <- data.frame( condition=sub( '\\.[0-9]', '', colnames(count.table) ))
rownames(condition)   <- colnames(count.table)

# -- Prepare DESeqDataSet
dds <- DESeqDataSetFromMatrix(countData = count.table, colData = condition,
                              design = ~ condition)

# -- Differential expression analysis ---------------------------------------------------

dds       <- DESeq(dds)
res       <- results(dds, contrast=c("condition","imp","rna"))
res.lfc   <- lfcShrink(dds, contrast=c("condition","imp","rna"), res=res)
de.result <- as_tibble(res.lfc)
de.result <- de.result %>% setNames(paste0('DESeq2.', names(.)))
de.result <- de.result %>% mutate(gene.id=rownames(res.lfc))

# -- Rank results by fold change (all) ---------------------------------------------------

de.result <- de.result %>%
  mutate(DESeq2.fc.rank=dense_rank(dplyr::desc(DESeq2.log2FoldChange)))

# -- Rank results by fold change (significant and positive) -----------------------------

de.targets     <- de.result %>% filter(DESeq2.padj<0.01 & DESeq2.log2FoldChange > 0)
de.target.rank <- de.targets %>%
  mutate(DESeq2.target.fc.rank=dense_rank(dplyr::desc(DESeq2.log2FoldChange))) %>%
  select(gene.id, DESeq2.target.fc.rank)

# -- Bring results together to main table  -----------------------------------------------

# -- attach subset to main (DE) table
de.result <- left_join(de.result, de.target.rank)

# -- attach bigger table to main (counts) table
gene.counts <- left_join( gene.counts, de.result )

# -- SAVE
save(gene.counts,
```

```
    file=file.path(project.home, target.dir, 'counts.ranked.with.DESeq.RData') )
```

Annotate overlapping genes

```
#-#-#-#-#-#-#-#-#-#-#-#-#-#-#-#-#-#-#-#-#-#-#-#-#-#-#-#-#-#-#-#-#-#-#-#-#-#-#-#-#-#-#-#-#-
# Indicate in Imp targets table which ncRNAs overlap other genes
#-#-#-#-#-#-#-#-#-#-#-#-#-#-#-#-#-#-#-#-#-#-#-#-#-#-#-#-#-#-#-#-#-#-#-#-#-#-#-#-#-#-#-#-#-

# -- Compare Imp targets table to genes.with.overlaps table -----------------------------

# -- Load info on gene overlaps (genes.with.overlaps) (see above)
load(file.path( project.home, '/Preprocessing/Genome/GeneSets/',
                'Dm.BDGP6.22.97.gene-overlap.info.RData' ))

# -- Load gene.counts object
load(file=file.path(project.home, target.dir, 'counts.ranked.with.DESeq.RData') )

# -- Indicate non-coding RNAs that overlap another gene
noncoding.ol <- genes.with.overlaps %>%
  dplyr::filter(query.gene.biotype != 'protein_coding') %>%
  dplyr::select(query.gene.id)
gene.counts <- gene.counts %>%
    mutate(is.noncoding.overlaps.another.gene =
             ifelse( gene.id %in% noncoding.ol$query.gene.id, 'yes', NA))

# -- Indicate non-coding RNAs that overlap a protein coding gene
noncoding.ol.coding <- genes.with.overlaps %>%
  dplyr::filter(query.gene.biotype != 'protein_coding' &
                  target.gene.biotype=='protein_coding') %>%
  dplyr::select(query.gene.id)
gene.counts <- gene.counts %>%
    mutate(is.noncoding.overlaps.coding.gene =
             ifelse( gene.id %in% noncoding.ol.coding$query.gene.id, 'yes', NA))

# -- Save
save(gene.counts, file=
       file.path(project.home, target.dir, 'counts.ranked.with.DESeq.with.overlaps.RData') )
```

GO term analysis

```
#-#-#-#-#-#-#-#-#-#-#-#-#-#-#-#-#-#-#-#-#-#-#-#-#-#-#-#-#-#-#-#-#-#-#-#-#-#-#-#-#-#-#-#-#-
# Which genes map to cellular growth, brain development, and regulator GO terms?
#-#-#-#-#-#-#-#-#-#-#-#-#-#-#-#-#-#-#-#-#-#-#-#-#-#-#-#-#-#-#-#-#-#-#-#-#-#-#-#-#-#-#-#-#-

go.dir <- file.path( target.dir, 'GO/' )
dir.create(file.path(project.home, go.dir))

# -- Define GO terms of interest --------------------------------------------------------

# -- Load GO terms from GO.db
```

```r
GOTERM.list <- as.list(GOTERM)
GO.id2term <- sapply(GOTERM.list, Term)

# -- Cell growth GO terms
GO.cell.growth <- tibble::enframe(GO.id2term[ grep('cell growth', GO.id2term) ]) %>%
   arrange(nchar(value))
write_tsv(GO.cell.growth, file.path(project.home, go.dir, 'GO.cell.growth.tsv'))

# -- Cell size GO terms
GO.cell.size <- tibble::enframe(GO.id2term[ grep('cell size', GO.id2term) ]) %>%
   arrange(nchar(value))
write_tsv(GO.cell.size, file.path(project.home, go.dir, 'GO.cell.size.tsv'))

# -- Cell division GO terms
GO.cell.division <- tibble::enframe(GO.id2term[ grep('cell division', GO.id2term) ]) %>%
   arrange(nchar(value))
write_tsv(GO.cell.division, file.path(project.home, go.dir, 'GO.cell.division.tsv'))

# -- Cell cycle GO terms
GO.cell.cycle <- tibble::enframe(GO.id2term[ grep('cell cycle', GO.id2term) ]) %>%
   arrange(nchar(value))
write_tsv(GO.cell.cycle, file.path(project.home, go.dir, 'GO.cell.cycle.tsv'))

# -- Brain development and neurogenesis GO terms
GO.neurogenesis <- tibble::enframe(GO.id2term[
  grep('(neurogenesis)|(nervous system development)', GO.id2term) ]) %>%
   arrange(nchar(value))
write_tsv(GO.neurogenesis, file.path(project.home, go.dir, 'GO.neurogenesis.tsv'))

# -- RNA-binding GO terms
GO.RNA.binding <- tibble::enframe(GO.id2term[ grep('RNA binding', GO.id2term) ]) %>%
   arrange(nchar(value))
write_tsv(GO.RNA.binding, file.path(project.home, go.dir, 'GO.RNA.binding.tsv'))

# -- DNA-binding GO terms
GO.DNA.binding <- tibble::enframe(GO.id2term[ grep('DNA binding', GO.id2term) ]) %>%
   arrange(nchar(value))
write_tsv(GO.DNA.binding, file.path(project.home, go.dir, 'GO.DNA.binding.tsv'))

# -- Nucleic acid binding GO terms
GO.na.binding <- tibble::enframe(GO.id2term[ grep('nucleic acid binding', GO.id2term) ]) %>%
   arrange(nchar(value))
GO.na.binding <- GO.na.binding %>% dplyr::filter( !grepl( "non-nucleic acid binding", value))
write_tsv(GO.na.binding, file.path(project.home, go.dir, 'GO.nucleic.acid.binding.tsv'))

# -- Map genes to GO  -----------------------------------------------------------------

# -- Get GO terms for all genes using biomaRt
mart.fly    <- useMart(biomart = "ensembl", dataset = "dmelanogaster_gene_ensembl")
gene.to.go <- as_tibble( getBM( attributes = c('ensembl_gene_id', 'go_id', 'name_1006'),
                                filters = 'ensembl_gene_id', mart = mart.fly,
                                values = gene.counts$gene.id))
```

```r
# -- Identify genes with GO terms of interest
cell.growth.genes   <- gene.to.go %>% semi_join( GO.cell.growth,   by = c('go_id'='name'))
cell.size.genes     <- gene.to.go %>% semi_join( GO.cell.size,     by = c('go_id'='name'))
cell.division.genes <- gene.to.go %>% semi_join( GO.cell.division, by = c('go_id'='name'))
cell.cycle.genes    <- gene.to.go %>% semi_join( GO.cell.cycle,    by = c('go_id'='name'))
neurogenesis.genes  <- gene.to.go %>% semi_join( GO.neurogenesis,  by = c('go_id'='name'))
RNA.binding.genes   <- gene.to.go %>% semi_join( GO.RNA.binding,   by = c('go_id'='name'))
DNA.binding.genes   <- gene.to.go %>% semi_join( GO.DNA.binding,   by = c('go_id'='name'))
na.binding.genes    <- gene.to.go %>% semi_join( GO.na.binding,    by = c('go_id'='name'))

# -- Load gene.counts
load(file.path(project.home, target.dir, 'counts.ranked.with.DESeq.with.overlaps.RData') )

gene.counts <- gene.counts %>% mutate(
  GO.cell.growth        = ifelse( gene.id %in% cell.growth.genes$ensembl_gene_id,   'yes', 'no' ),
  GO.cell.size          = ifelse( gene.id %in% cell.size.genes$ensembl_gene_id,     'yes', 'no' ),
  GO.cell.division      = ifelse( gene.id %in% cell.division.genes$ensembl_gene_id, 'yes', 'no' ),
  GO.cell.cycle         = ifelse( gene.id %in% cell.cycle.genes$ensembl_gene_id,    'yes', 'no' ),
  GO.neural.development = ifelse( gene.id %in% neurogenesis.genes$ensembl_gene_id,  'yes', 'no' ),
  GO.RNA.binding        = ifelse( gene.id %in% RNA.binding.genes$ensembl_gene_id,   'yes', 'no' ),
  GO.DNA.binding        = ifelse( gene.id %in% DNA.binding.genes$ensembl_gene_id,   'yes', 'no' ),
  GO.nuc.acid.binding   = ifelse( gene.id %in% na.binding.genes$ensembl_gene_id,    'yes', 'no' ))

# -- Save
save(gene.counts,
     file=file.path(project.home, go.dir,
                    'counts.ranked.with.DESeq.with.overlaps.with.GO.RData'))
```

Make Figure 2

```r
#-#-#-#-#-#-#-#-#-#-#-#-#-#-#-#-#-#-#-#-#-#-#-#-#-#-#-#-#-#-#-#-#-#-#-#-#-#-#-#-#-#-#-#-#-#-
# Make a two-panel figure
#-#-#-#-#-#-#-#-#-#-#-#-#-#-#-#-#-#-#-#-#-#-#-#-#-#-#-#-#-#-#-#-#-#-#-#-#-#-#-#-#-#-#-#-#-#-

# -- Select top 40 ranked genes that are not ncRNAs that overlapping with other genes  --

# -- Load gene.counts
load(file.path(project.home, go.dir, 'counts.ranked.with.DESeq.with.overlaps.with.GO.RData'))

# -- Select top
data <- gene.counts %>% dplyr::filter(is.na(is.noncoding.overlaps.another.gene)) %>%
  dplyr::slice(1:40)

# -- Panel A  --------------------------------------------------------------------------

# -- Get imp over RNA ratio
data.pop <- data %>%
  dplyr::select(gene.id, gene.name, imp.over.rna.lib.mean )

# -- Remove 'lncRNA' from lncRNA names as it makes their names very long
data.pop <- data.pop %>%
  dplyr::mutate(name=sub('lncRNA:', '', gene.name)) %>%
```

```r
  dplyr::mutate(gene=factor(name, levels = name))

# -- Highlight Myc (FBgn0262656) and Mnt (FBgn0023215) in the plot
goi <- c('Myc', 'Mnt')

# -- Plot
panel.a <- ggplot(data.pop, aes(x=gene, y=imp.over.rna.lib.mean)) +
  geom_segment( aes(x=gene, xend=gene, y=0, yend=imp.over.rna.lib.mean ),
                color="grey", size=0.15) +
  geom_point( color= ifelse(data.pop$gene %in% goi, "orange", "grey"), size=1.3 ) +
  theme_minimal() +
  scale_x_discrete(breaks = NULL) +
  theme(axis.text.x=element_blank(), axis.title.y=element_text(size=10),
        panel.grid.major= element_line(size = 0.15, linetype = 'solid'),
        panel.grid.minor = element_line(size = 0.15, linetype = 'solid') ) +
  labs(title="Top Imp targets", x = "",
       y = "Imp RIP-seq / RNA-seq \n(normalised read counts)" )  +
  annotate("text", x=data.pop$gene, y=data.pop$imp.over.rna.lib.mean+0.20,
           label=data.pop$gene, color=ifelse(data.pop$gene %in% goi, "orange", "black"),
           size=2.5, angle=90, fontface="bold.italic", hjust=0)

panel.a
```

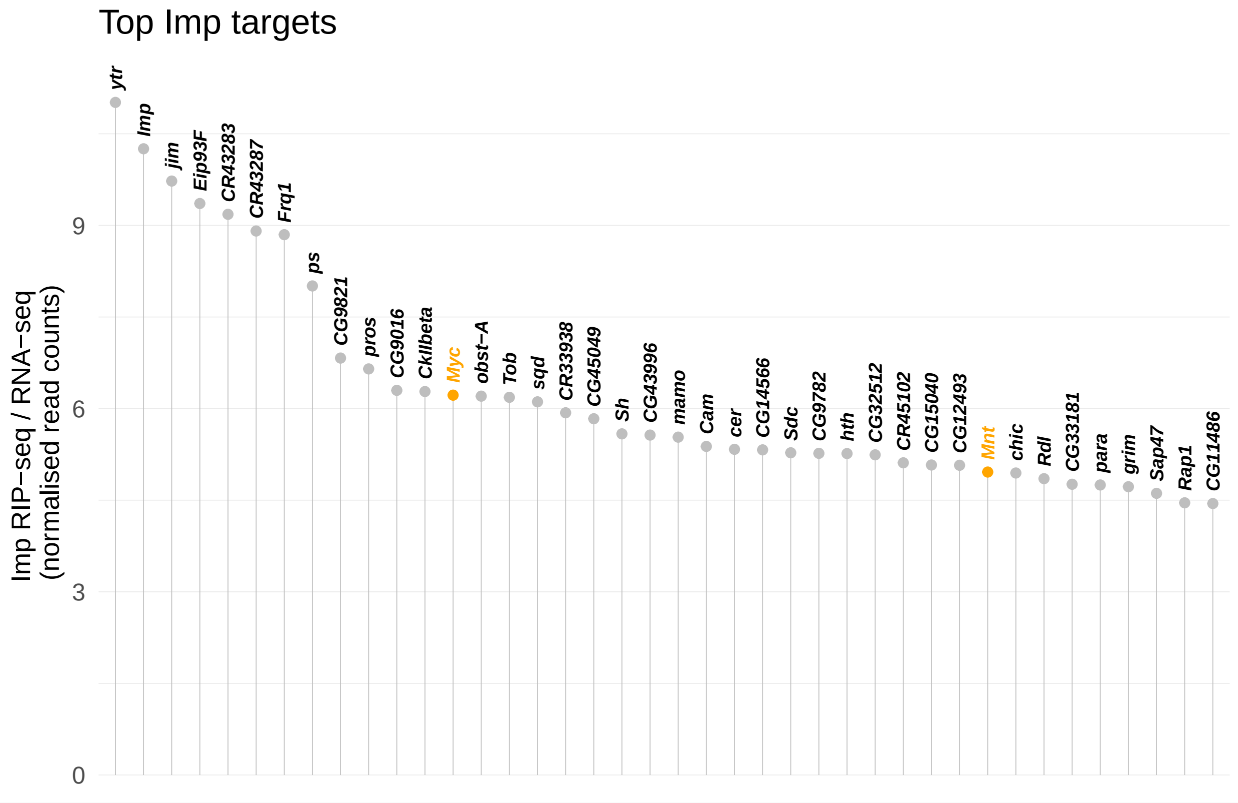


```r
# -- Panel B  -------------------------------------------------------------------------

# -- Prepare genes & GO terms in a format suitable for a cleveland plot
data.go    <- data %>%
  dplyr::select(gene.id, gene.name, starts_with('GO'), -GO.nuc.acid.binding)
go.coord   <- tibble( term=names(data.go)[grep( 'GO', names(data.go) )],
```

```
                    pos=length(grep( 'GO', names(data.go) )):1 )
go.coord  <- go.coord %>% mutate(name=gsub( '\\.', ' ', sub('GO.', '', term) ))
data.long <- data.go %>% gather(starts_with('GO'), key=term, value=has.term)
data.long <- left_join(data.long, go.coord) # problem probably comes from here.
data.long <- data.long %>% mutate( pos.plot = ifelse(has.term=="yes", pos, NA))
data.wide <- data.long %>% spread( key=term, value=pos.plot )
data.wide <- data.wide %>% mutate( name=factor(gene.name, levels=data.go$gene.name ) )

# -- Add extra 'genes' (NA) to make the grid wider to make space for GO labels
nextra   <- 5
nterms   <- data.go %>% dplyr::select(starts_with('GO')) %>% names() %>% length()
anno.row <-  tibble(gene.id=rep(paste("label", 1:nextra),each=nterms),
                    gene.name="",
                    name=rep(paste("label", 1:nextra),each=nterms),
                    has.term="no",
                    pos=rep(1:nterms, nextra),
                    GO.cell.cycle=NA, GO.cell.division=NA, GO.cell.growth=NA,
                    GO.cell.size=NA, GO.DNA.binding=NA, GO.neural.development=NA,
                    GO.RNA.binding=NA)
data.wide.2 <- bind_rows(data.wide, anno.row)

# -- Remove 'lncRNA' from lncRNA names as it makes their names very long
data.wide.2 <- data.wide.2 %>% mutate(
  name=factor(sub( 'lncRNA:', '', data.wide.2$name),
              levels=c( sub( 'lncRNA:', '', levels(data.wide$name) ),
                        paste("label", 1:nextra) )))

# -- Assing a colour to points based on how many GO terms the gene maps to
hits.per.gene <- data.wide.2 %>% group_by(gene.id) %>%
  summarise(hits=length(which(has.term=='yes') ))
cols <- tribble(
  ~hits, ~point.colour,
  4, '#000080',
  3, '#00008090',
  2, '#00008070',
  1, '#00008040'
)

hits.per.gene <- left_join(hits.per.gene, cols)
data.wide.2 <- left_join(data.wide.2, hits.per.gene)

# -- Highlight Myc and Mnt in orange
col <-  c( rep("black",40), rep(NA,nextra))
col[data.go$gene.name %in% c('Myc', 'Mnt')] <- "orange"

# -- Plot
go.grid <- ggplot(data.wide.2) +
  geom_point( aes(x=name, y=GO.cell.cycle ), size=3, colour=data.wide.2$point.colour ) +
  geom_point( aes(x=name, y=GO.cell.division ), size=3, colour=data.wide.2$point.colour ) +
  geom_point( aes(x=name, y=GO.cell.growth ), size=3, colour=data.wide.2$point.colour ) +
  geom_point( aes(x=name, y=GO.cell.size ), size=3, colour=data.wide.2$point.colour ) +
  geom_point( aes(x=name, y=GO.neural.development ), size=3, colour=data.wide.2$point.colour ) +
  geom_point( aes(x=name, y=GO.DNA.binding ), size=3, colour=data.wide.2$point.colour ) +
```

```
  geom_point( aes(x=name, y=GO.RNA.binding ), size=3, colour=data.wide.2$point.colour ) +
  theme_minimal() +  xlab("") + ylab("") +
  theme(axis.text.x=element_text(angle=90, hjust=1, vjust=0.5, face="italic", colour = col ),
        axis.text.y=element_blank())

panel.b <- go.grid +
  theme(panel.grid.major = element_blank(), panel.grid.minor = element_blank()) +
  geom_vline(xintercept=1:40,  color = "#00000080", size=0.05) +
  geom_hline(yintercept=2.5, color = "#FFFFFF", size=2) +
  geom_hline(yintercept=2.5, color = "#00000080", size=0.15, linetype="dashed") +
  annotate("text", x = 41, y = go.coord$pos, label=go.coord$name, hjust=0, size=3 )

panel.b
```

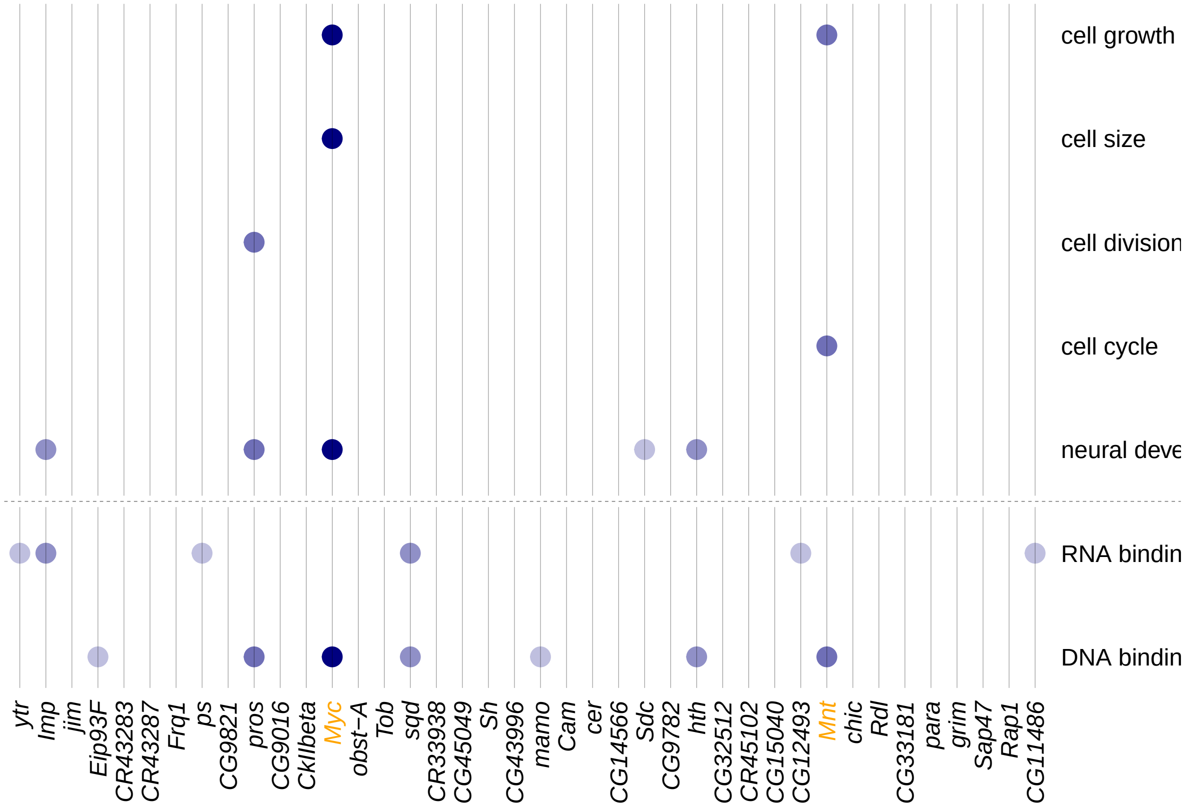

Supplement: Source code 1. — Details and code used for the bioinformatic analysis of the Imp RIPseq and RNAseq data presented in Figure 2 and Figure 2—figure supplement 1. [file elife-51529-code1.pdf]
