# Supplementary material for: Imp/IGF2BP levels modulate individual neural stem cell growth and division through myc mRNA stability
Source: eLife. 2020 Jan 14;9:e51529. doi: 10.7554/eLife.51529 (PMC7025822; doi:10.7554/eLife.51529)
Supplement: Supplementary file 2. [file elife-51529-supp2.docx]

Stellaris Probes

| ***myc* exon - Quasar 670** | ATGGCCCTTTACCGCTCTGATCCGTATTCCATAATGGACGACCAACTTTTTTCAAATATTTCAATATTCGATATGGATAATGATCTGTACGATATGGACAAACTCCTTTCGTCGTCCACCATTCAGAGTGATCTCGAGAAGATCGAGGACATGGAAAGTGTATTTCAAGACTATGACTTAGAGGAGGATATGAAGCCAGAGATCCGCAACATCGACTGCATGTGGCCGGCGATGTCCAGCTGTTTGACCAGCGGTAACGGTAATGGAATAGAGAGCGGAAACAGTGCAGCCTCGTCGTACAGCGAAACCGGTGCCGTATCCCTGGCGATGGTTTCCGGCTCTACGAATCTCTACAGCGCGTATCAACGATCGCAGACGACAGATAACACCCAGTCAAATCAACAGCATGTCGTCAACAGTGCCGAGAACATGCCGGTGATCATCAAGAAGGAGCTCGCAGATCTGGACTACACGGTCTGTCAGAAGCGCCTCCGTTTGAGCGGCGGTGACAAGAAGTCACAGATCCAGGACGAGGTCCATTTAATACCGCCCGGCGGAAGTTTGCTCCGCAAGCGGAACAACCAGGACATTATCCGCAAATCGGGCGAATTGAGCGGCAGCGATAGCATAAAATACCAGAGACCAGACACACCTCACAGTCTTACCGACGAGGTGGCCGCCTCAGAGTTTAGACATAACGTCGACTTGCGTGCCTGCGTGATGGGCAGCAATAATATCTCGCTGACCGGCAATGATAGCGATGTCAACTACATTAAGCAAATCAGCAGGGAGCTTCAGAATACCGGCAAGGATCCGTTGCCGGTGCGTTACATCCCGCCGATCAACGATGTCCTCGATGTGCTCAACCAGCATTCCAATTCGACGGGTGGCCAACAGCAGTTGAACCAACAGCAACTGGACGAGCAACAACAGGCCATCGATATAGCCACTGGACGCAACACAGTGGATTCTCCGCCGACGACCGGCTCTGATAGTGACTCCGATGACGGTGAACCCCTCAACTTTGACCTGCGCCATCATCGCACTAGCAAAAGCGGCAGCAATGCCAGCATCACCACCAACAACAACAACAGCAACAACAAAAACAACAAATTGAAGAACAACAGCAACGGCATGCTGCACATGATGCACATCACCGATCACAGCTACACGCGCTGCAACGATATGGTGGACGATGGTCCCAATTTGGAGACCCCCTCAGATTCCGATGAGGAAATCGATGTCGTTTCATATACGGACAAGAAGCTACCCACAAATCCCTCGTGCCACTTGATGGGCGCCCTACAGTTCCAGATGGCCCATAAGATCTCGATTGATCACATGAAGCAAAAACCGCGCTACAATAACTTCAATCTGCCGTACACACCGGCCAGCAGCAGTCCAGTGAAATCGGTGGCCAACTCGCGTTATCCATCACCGTCGAGCACACCGTATCAGAACTGCTCCTCCGCTTCGCCGTCCTACTCGCCGCTATCCGTGGACTCTTCAAATGTCAGCTCGAGCAGCTCCAGTTCCAGTTCGCAGTCAAGCTTCACCACCTCCAGTTCGAACAAGGGACGCAAACGATCCAGTCTGAAGG |
| --- | --- |
| ***myc* intron - Quasar 570** | AGAGGGAAACTACATTAAAAAAAAGTCCAGCTAGAATACGTGTTTTTCATATCTATATTTTTATAGTACTCCTACCTACTTGTTTTATTTGTGCCGGCGAGTTCAATGTCTGGCTCTCTTTCTCTCTTTTTGGCGCGCAAATCGTTATCCATTGAGCAACCCTCGCAAGCTTACGTCACGTCAATCATCTTGATTTTTACTACGTCTTACGCTACAACTACAACTGCGCATGCAACTACGTGTGCATTTTCTACTACAATGACTACTACAACTACAACTACTACTACTACTACGCTATGGAGCCGGTGTGGGCGTAAGCTTTTTGCCGCCGCTCATTAAAAAAAATATATGGTAAATAATAGCGGGAAATCAAGAAGGGGCTAGAGGTGGGGCAATGCAAGGGATAAATTAAACTTGGATTATAAAATATTTACCAATAAAGCAAAAAGCAAGAGCAACAGCAAGAGCAAGAGCAAAAGCATTTAACTCGTGTTTCTGCTCCCGCTTTTTCTCTCTCGCTCGCCTGTCTTTAGTTTCTCTCTTGCAATTTGCCGAGTGCAGTTAAACCGAGAAATTGCAGTAACAACAAGTTTGTTTTGTACGTTTGTTTTGGTTTATATGCTTTGCTTTTTTCTGCGCTCTGCTTTTTGGGTGTTGTCTGCTTTTGCGTTTTGCGTGCTTTTTTTTTTGTGTGTTTTATGCTTTTGCTTCTACCGCAACGAACGGAACGCAAAAAATGTCAAAAAAGAAAATGTGCAATATGTAAAATGCTTACCATAAATTGAGGAAAACCCACACACGCGTGTGTATGAATTTTAATTACTCAGAATCAGAAAGACAAATTTACGTAGAATTGCTAGTATTTTATGATCGTTTGGCAAAATGGAATTCTGAAGCGTTATCATCATCTTAGTTTGGTGACGAAATCGCATCATAATTTTCCACTGCTAATTTGGCAAACTAAAAGCTTGACACAAGTGGCTCGAGATTAGTTCTTTCTATTCTTTTTGCGTGCGCGATTCTTGTTGGAAGCAGATATGGGAATAGCAGAAGAAACGTAAAAAGAAATCAGGTGCGTGCCGCAGATTAGCAAATCTTTGCCCTCTTGCTCGCACGCGAAACTCGTGAGGCTTGCGAGAGGGAGATGGCGCTCTTCGAACACACGCACTTGTATCAATATCAATTCTTTTCTTTCCTTTATTGTGGAGGGGGGTTGCTTTCTTTATCGCTATGCTGCAGTCTTCTAATTAGTATAAAAGCTTACGTGAGCGAGCGAGACGGGGCGCGTCACGTGCGAGCGAGAGCGGCGGTTGGCAACAACGCACGAAATTCGGCACATAGATAAGGTTCACGGGGGAGTGAGCGAGAGACTAGAGGCCACATGCCTATGATGTTGGCGCCTCTATAAAAGTGCGTACACACACACACACACATACATGACTGGCTGTGTACGTATAATATGTATATGTATTTACCTTTGGCAGTGCGTGCTGGTTTTTGGCATTTCGCACCTTGCGACTGTGTGTGTGTGTGTGCGTTTGTGGGTTTGGAAAAATGTTAATGAACTGAACCAAGGTCAGCCATTCGCCGGAAAAAAAAGTGCGTAGAAACCGAAAATTAGTGGTGTGGGGGTGCGGCACAGAAAAAAAAAAAAAAAAAAACGTATGGCCAAGTCATGTTGGCTAAAACAATATTTCCTATTTTGGCCTCTTCGCTTTACACTTTCGATCTCAGCAGCTCTTCTGGGCTCTCTTGTGGCCTTTTAGAAGAAGACAACAATCGAAAGTATAGAGACATGTAAACAACATTAAATGAAATTAAATAACGCCCAAGAAGAAAGAAGAAACTGTATGAAAAAGTAGTGGATAATAATAATACGAAAACCCCAAAGACTAGAACATAAATAGTTGTCGATTGTTG |
| ***myc* 3’ UTR - Quasar 570** | AGCAAGAAAATTTCTATAGTGTAGGGCGCGAGACGTTTCAACGAAAATTTTTTTCTATGTGAGTTCTAGGCTAATGCTGCCTACTAAACCTATACCCAGATATACATATAATTCTTATATTTTTGTTTACCTAGTAGCTTAACGTTCGAACACATGAAATATTTTTTACCTTATACATTTCTTTAAGTACGCCGCCGCAAAAACTTTTGAAAAACTACCTACTTAACTCTTCATTTTTGTAATAAAAAGAAAAAAAAACAACAAACAAACAACAACAACTATAAAACAATTGTTAAATGGAAAATAATGATATATATCTATCTACGTGGACAAACAATTAAAGAGTATATTTTTTGTATTGTTTCGACCAAAAAATGTGTACACACTGTAAATAGCAATCTCGTTAAATAATAGTTACACACGCCTACAAAATAGCGAACCCAAAACCCAAGAAATGGAATTATTTTTATAGAAAACAAAAAAACCCGGAGAGAAAACACAAAGCAATAGACTTAAGCGAATTGTACAACGCGAAACGAAAACAAAACTTCAATAAACCAACAAAAACACACACACTTATATATATATACATATATATATGTATATATACATATACAAAACCACACACGAATGCACCTATTTTCCTATAGTACATACAACCAGAAATAGTTAAACGAAAAAACCATGTTTTCTTTCAATAATTTCAACAAACAAAACCGTACAAATTTACAAGAAACAAAAATACGAAAAGCAAACCTTTTCTTTGTTTGGTCCTTTTATTAATTTATTAACGAAACAACAAATTCAAGTGAAAGGGCATTTTTAAACATAATTTTTCATTGTAAAAAAAACAAAATATATACACTAAAACTATGACCAAAACCAAAATCCTCGCAAACAAAACAAAAAAATATTAAATTTTTTTCTTTTAAAATATATTTATAACAAAAAACAAAACAAAAGTTTTTAAGTTTAAATATATTTTTATGATTATCAAATTTTTTATATTATATACACACACACAAACTACTATTTGAATTAGTTGTTAAAAAATTTATATTATTAAAACAACACATTATTTGAAGACAAATAACATAAAAAAAAAACTATGTAAAAAAAAATCTGAAAAACTCATGAAATGAAAGCAAAAAACTGTTAAAGCCGTACGGAAAATATGAAACTATAGACGAACATGCTTGAATTATTACATGTATATTTAAATTAATTTTTTTTTTTAGTCATAAACGTATGCAAAAAACGTATACGTTTATGGCTATGCATTTGAAATCCCTATTTTTGATTTGATGACAAAAGAAGATATGAAAATTCTGTTTACTTTGGTTATTGCTTATTAAATATGTGTAAAAATTGCAAAATAATATAGATATATCTGGCAAAACAAGCTAAACAACTATATTAATATATTATCTAATAAATTTTAACTCGTTATGTAGTTACCTATTAATGAAAAACAAATTAAAAGCAAAAAAAATGAGAAAAGTAAACACAATAAATTACATTTTATGTACCTTCTACATATATAAACTAAATATATTACACAGAACACACACACTTATATATAAACACACACACACACACACACACTACTATATATAGAAACCGTTTAAATATTTTTTTTTTCAAAATTTCTGATAATGGAATTATATATACATTATACAATAAAAATACTTCACCAACAAATTACTTTTGATTTTGTTTTTATTTTTACGCTTGATTCCTTTTCGTTTAATATATGGATGCAATTATTGAAGCTTCCCAGATCTTTCAATTGGCACAAGGATTTCCTCTTGTTATTTGCGTTTTCGTATTCGAATATTCAAATATTCGAATATGCAATTTTGTTGCACATTTTTTCTGGACGCCCTGTTTTATTTTATTTACTTTCAGCGACTTTCAAAACCCCAAGTGTGTTTCATCCGAGTTGTTTATATTTGTTTTACTTTAGAAAATCGTATTGTGTCTCACAATAAGCTGGCTAAAAAAAAACAGCTTTTCCTTTTTTGGAATTTGGTACTATCCAACTTGTGCTTCACTTTCATTTTCCAACCGCGCCGAGTATCAAATTCGCAGTGAATCATTCGAGTGAGTAAGTGATTCACATTTGTGGGTGGTGGGAAAATTGCTTTTGTCGCCGTTTCTAATCTAGCAGTTCAACGTATTTTTTCTCGTTGCCTGGTTGCGCTGCCTCGCTCATTTGCCCACCAGACAAATTAAGTTAATTCAATAAGAGTTGTTGCTCACTATTGTTGTTGTTGTTTTACCGGCAAATTTTTTGTTTTTAATGAAATCAAAGCGACAACAACGACAGCCGAGAAGCCGGTTGGGATACAGTCAGCGACATGACGACAATAACAACGATCACAACGATAGCCATATAATATAACATTTTATTTATATGTACGTGTTTTGTTTTCGTTGATTTTTGCTGGTTTATTATTTTACTACAATCATTCAGTTACGGTTTCGTTGGTTGAATTTTTTTCTTTTAATTTTTACTCGAAACTTTCGGAAGAAACCAAAACGAAATAGGCGATGCTCTTATTTGAATGTACAATATATTATTTAAACAGTGTGTGTTGTATAGTGTTTTATGCGTGTTCTATGAAAAGTGTTGTTAATTGTTTGGATATATTTTTTAAGCATCACATAAAGTTTTCTGTTGGGTCGTTTAAGTTTAAAAGTTTAAAATGGAGAGCTAAAACAGATGAATTAAACTTAATTTGCAGTTTATTATGACAGTGGGGATACCGCGTTAAATAGAAAGGGTACACGAATGGCAGAAGTAGCCGGCAAATTCACACACACACACACACACACAGCGGGCCAAGCAAAACAGAGACAACGAAATTGGGGTGCACA |
